# Tillage, Mulch and N Fertilizer Affect Emissions of CO_2_ under the Rain Fed Condition

**DOI:** 10.1371/journal.pone.0072140

**Published:** 2013-09-25

**Authors:** Sikander Khan Tanveer, Xiaoxia Wen, Xing Li Lu, Junli Zhang, Yuncheng Liao

**Affiliations:** College of Agronomy, Northwest A&F University Yangling, Shaanxi, P.R. China; DOE Pacific Northwest National Laboratory, United States of America

## Abstract

A two year (2010–2012) study was conducted to assess the effects of different agronomic management practices on the emissions of CO_2_ from a field of non-irrigated wheat planted on China's Loess Plateau. Management practices included four tillage methods i.e. T_1_: (chisel plow tillage), T_2_: (zero-tillage), T_3_: (rotary tillage) and T_4_: (mold board plow tillage), 2 mulch levels i.e., M_0_ (no corn residue mulch) and M_1_ (application of corn residue mulch) and 5 levels of N fertilizer (0, 80, 160, 240, 320 kg N/ha). A factorial experiment having a strip split-split arrangement, with tillage methods in the main plots, mulch levels in the sub plots and N-fertilizer levels in the sub-sub plots with three replicates, was used for this study. The CO_2_ data were recorded three times per week using a portable GXH-3010E1 gas analyzer. The highest CO_2_ emissions were recorded following rotary tillage, compared to the lowest emissions from the zero tillage planting method. The lowest emissions were recorded at the 160 kg N/ha, fertilizer level. Higher CO_2_ emissions were recorded during the cropping year 2010–11 relative to the year 2011–12. During cropping year 2010–11, applications of corn residue mulch significantly increased CO_2_ emissions in comparison to the non-mulched treatments, and during the year 2011–12, equal emissions were recorded for both types of mulch treatments. Higher CO_2_ emissions were recorded immediately after the tillage operations. Different environmental factors, i.e., rain, air temperatures, soil temperatures and soil moistures, had significant effects on the CO_2_ emissions. We conclude that conservation tillage practices, i.e., zero tillage, the use of corn residue mulch and optimum N fertilizer use, can reduce CO_2_ emissions, give better yields and provide environmentally friendly options.

## Introduction

Studies regarding soil CO_2_ emissions have attracted significant attention because the concentration of CO_2_ in the atmosphere is increasing very rapidly as a consequence of fossil fuel combustion and deforestation. The past two centuries of human activities have reportedly contributed as much as approximately half of the increase in CO_2_ emissions [1], [2]. Global terrestrial ecosystems absorbed carbon at the rate of 1–4 Pg yr^−1^, during 1980s and 1990s, which made up approximately 10–60% of the fossil fuel emissions [3], [4]. Currently, significant attention is given to CO_2_ emissions from soils because this source significantly affects the global carbon cycle and the function of the terrestrial ecosystem [5]. Fluxes of greenhouse gases (CO_2_, N_2_O and CH_4_) between the atmosphere and agricultural soils considerably influence the stock of anthropogenic greenhouse gases [6]. Agriculture is an important source of emissions for these different gases, and its contribution to climate change is approximately 20% on an annual basis [7]. It has been reported that soils have already contributed approximately 50 Pg of anthropogenic CO_2_ to the atmosphere in the past, through cultivation processes [8].

Tillage is an integral part of agriculture which not only significantly affects crop production but is also considered one of the leading factors in soil degradation. This technique is a fundamental operation that has affected both the soil and the environment and is considered one of the most important sources of CO_2_ emissions into the atmosphere [9] because humans have tilled the soil for crop production for thousands of years [10] and approximately 23–44% of total CO_2_, is emitted into the atmosphere through soil preparation-related operations [11]. Approximately 30–50% of soil C has already been lost through the adaptations of intensive tillage practices [12], and major C losses from, soils in the form of CO_2_ occur immediately after the tillage operations [13].

Agricultural management practices affect different soil processes (i.e., soil temperature, soil moisture and soil pH), and other ongoing soil decomposition processes, which ultimately result in the conversions of plant-derived C to soil organic matter and CO_2_ [14]. Applications of inorganic as well as organic fertilizers [15] and different degrees of soil moisture and temperature strongly affect the fluxes of soil CO_2_ [16], [17] & [18]. Similarly, the application of N fertilizer also affects soil CO_2_ emissions [19]. Instead of burning crops residues, farmers, applications of inorganic fertilizers and use of green manures as well as organic manures can be of great use in maintaining soil fertility [20]. These practices can provide essential nutrients to crops and reductions in the burning of crops can reduce CO_2_ emissions into the atmosphere [21].

Agricultural tillage practices can be helpful in the sequestering of atmospheric CO_2_ [22], [23] and [24]. Conservation tillage has the potential to increase soil C and N [25] and other types of conservation practices can be helpful in reducing the loss of soil organic carbon from the soils [26], [27]. Similarly, the retention of crop residue, nitrogen fertilization and no-tillage are generally supposed to enhance the soil organic carbon (SOC) stocks in the soil [28] because these farms, management practices not only increase crops biomass, but are also considered very important for the microbial decomposition of crop residues [29]. As far as N fertilization is concerned, some scientists have reported that increased N fertilization can depress CO_2_ emissions [30], [31] however, others [32] have reported that N fertilization has no effect on SOC, while some other scientists [33] have reported that higher N fertilization improves the SOC of the soil.

The Loess Plateau has an area of approximately 63, 5000 km^2^. It covers many provinces in China, and is home to millions of people. It is one of the most highly eroded areas of the world, and traditional agriculture, i.e., intensive tillage, is considered one of the leading man-made factors responsible for this erosion. However many crops residues are produced in this region. A small portion of these residues is used for forage or fuel consumption and the remaining residues are generally burned. Mold board plowing followed by harrowing is commonly used for the tillage operations in this region [34]. Few studies showing CO_2_ emissions to the adaptation of different agronomic management practices have been previously reported from this region of China. In this area intensive tillage methods i.e. rotary tillage and mold board plow tillage methods are commonly used for land preparation. Commonly higher levels of N fertilizers are applied and crop residues are removed from the fields at the time of soil preparation.

The main aim of this two year study was to identify the effects of different tillage methods i.e. chisel plow and zero tillage in comparison with intensive tillage practices i.e. rotary tillage and mold board plow tillage methods, different N fertilizer levels and the application of corn residue mulch on CO_2_ emissions. The results from this study can be of great help in improving the management of soils not only in this area of China but also in other regions of the world.

## Results

### Wheat crop yields

**Figure 1:**
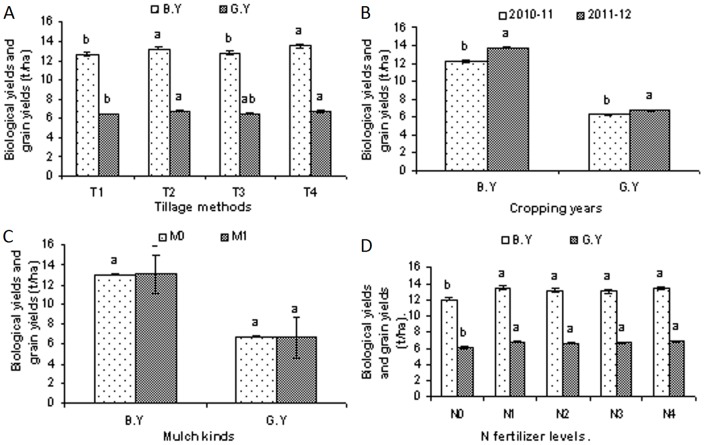
Wheat crop biological and grain yields as affected by different tillage methods, different mulch kinds and different N fertilizer levels during different cropping years (2010–12). (A), Wheat crop biological and grain yields as affected by different tillage methods i.e. T_1_, chisel plow tillage., T_2_, zero tillage., T_3_, rotary tillage and T_4_, mold board plow tillage., (B), Mean wheat crop biological and grain yields as affected by different tillage methods, mulch kinds and different N fertilizer levels during two cropping years (2010–12). (C), Wheat crop biological and grain yields as affected by different mulch kinds i.e. M_0_, No mulch and M_1_, corn residue mulch., (D), Wheat crop biological and grain yields as affected by different N fertilizer levels during two cropping years (2010–12) including, N_0_, 0 kg N/ha., N_1_, 80 kg N/ha, N_2_, 160 kg N/ha., N_3_, 240 kg N/ha and N_4_, 320 kg N/ha.

Significant variations in wheat crops biomass and grain yields were recorded during both study years (2010–12). When compared to the other tillage methods, there were better yields overall with the zero tillage planting method. No grain yields differences were recorded under different mulch treatments and similarly low yields were recorded for N_0_ nitrogen fertilizer levels. Statistically equal grain yields were recorded for all of the other higher tested levels of N fertilizer (Fig. 1).

### Soil CO_2_ flux

**Figure 2:**
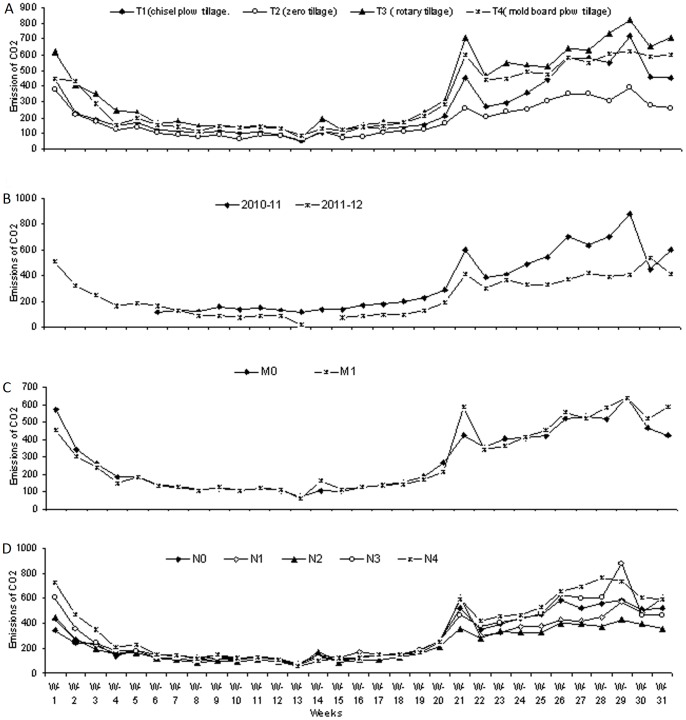
CO_2_ emission trends as affected by different tillage methods, mulch kinds and N fertilizer levels during two cropping years (2010–12). (A). Emissions trends of CO_2_ from different tillage methods i.e. T_1_, chisel plow tillage, T_2_, zero tillage, T_3_, rotary tillage and T_4_, mold board plow tillage., (B).CO_2_ emission trends as affected by different kinds of tillage methods, different types of mulch and different N fertilizer levels, during two cropping years (2010–12). (C). CO_2_ emission trends due to different mulch kinds i.e. M_0_, no mulch and M_1_, corn residue mulch., (D). CO_2_ emissions trends due to different N fertilizer levels during two cropping years (2010–12) including, N_0_, 0 kg N/ha., N_1_, 80 kg N/ha, N_2_, 160 kg N/ha, N_3_, 240 kg N/ha and N_4_, 320 kg N/ha.

**Figure 3:**
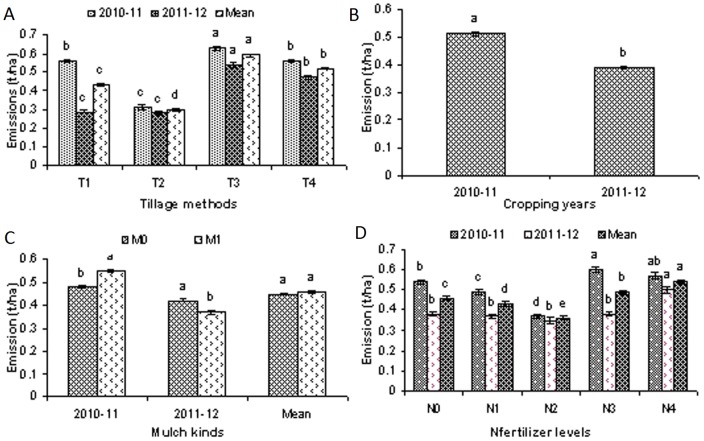
Total/mean emissions of CO_2_ as affected by different tillage methods, mulch kinds, and N fertilizer levels during two cropping years (2010–12). (A).Emissions of CO_2_ from different tillage methods i.e. T_1_, chisel plow tillage., T_2_, zero tillage.,T_3_, rotary tillage and T_4_., mold board plow tillage., (B). Total emissions of CO_2_ as affected by different kinds of tillage methods, different kinds of mulch and different levels of N fertilizer, during two cropping years (2010–12)., (C), Emissions of CO_2_ from the different mulch kinds i.e. M_0_, no mulch and M_1_, corn residue mulch., (D).Total/mean emissions of CO_2_ as affected by different levels of N fertilizer, during two cropping years (2010–12) including, N_0_, 0 kg N/ha., N_1_, 80 kg N/ha., N_2_,160 kg N/ha., N_3_, 240 kg N/ha and N_4_, 320 kg N/ha.

All tested treatments had significant effects on the CO_2_ emissions (Figs. 2, 3). The details are given below.

### Tillage method effects on CO_2_ emissions

Two years of combined data show that the rotary tillage and mold board plow tillage methods had their highest and statistically equal CO_2_ emissions during the first week of planting the wheat crop, compared to the chisel plow tillage and zero tillage planting methods. When compared to all the other tillage methods, the lowest CO_2_ emissions were recorded for the zero tillage planting method (Fig. 2; Fig. 3). Emission trends recorded during the whole wheat crop seasons show that although there were variations in the CO_2_ emissions in response to different tillage methods, the overall highest emissions were recorded for the rotary tillage planting method, followed by mold board plow tillage. Although higher CO_2_ emissions were recorded for the chisel plow tillage method, the lowest CO_2_ emissions were generally recorded for zero tillage planting method (Fig. 2; Fig. 3).

### Effects of cropping years on CO_2_ emissions

With the exception of one week (i.e., week 23), the highest weekly emissions of CO_2_ were recorded during cropping year 2010–11 in comparison to the weekly emissions of CO_2_ during cropping year 2011–12 (Figs. 2, 3).

### Mulch effects on CO_2_ emissions

Weekly CO_2_ emissions during both wheat crops growing seasons (2010–11 and 2011–12) and the mean of the two years (2010–12) of data (Figs. 2, 3) show that there were more CO_2_ emissions recorded from the corn residue-mulched treatments during cropping year 2010–11 than from the non-residue mulched treatments (Figs. 2, 3). However, fewer CO_2_ emissions were recorded from the corn residue mulched treatments during cropping year 2011–12 and in comparison to the non residue-mulched treatments, over all less emissions of CO_2_ were recorded from the corn residue mulched treatments (Figs. 2, 3). However the two year mean data show mixed types of emissions were recorded on a weekly basis following the applications of corn residue mulch or no mulch (Fig. 2).

### N fertilizer level effects on CO_2_ emissions

CO_2_ emissions data recorded during both wheat cropping seasons, i.e., 2010–11 and 2011–12 and the two year (2010–12)) mean data show that there were significant differences in the weekly CO_2_ emissions in response to different N fertilizer levels (Fig. 2; Fig. 3). This finding also indicates that the lowest CO_2_ emissions were recorded for the N_0_, N fertilizer level at the start of the wheat crop growing seasons compared to all the other higher N fertilizer levels (Fig. 2). During the winter months the CO_2_ emissions decreased under all treatments but when the temperatures rose again, higher CO_2_ emissions were recorded (Fig. 2). However, when compared to all the other N fertilizer level treatments, the lowest overall CO_2_ emissions were recorded for 160 kg N/ha (Fig. 2). CO_2_ emission fluxes increased with the increase in crop growth and temperatures, so during the last weeks of wheat crop growth equal CO_2_ emissions were recorded for all of the N fertilizer treatments (Fig. 2).

### Cumulative CO_2_ emissions

Two years (2010–12) of CO_2_ emissions data show that on a mean cumulative basis, except in the case of corn residue mulch treatments, significant differences in the emissions of CO_2_ were recorded for all of the different tillage methods and different N fertilizer level treatments (Fig. 3). Statistically significant variations in the total and mean CO_2_ emissions were recorded for all of the tillage methods, and the emissions trend for the different tillage methods was T_3_>T_4_>T_1_>T_2_ (Fig. 3). Different N fertilizer levels had significant effects on the total and mean CO_2_ emissions. The emissions trend for the different nitrogen fertilizer levels was N_4_>N_3_>N_0_>N_1_>N_2_ (Fig. 3). On a cumulative basis, more CO_2_ emissions were recorded during the cropping year 2010–11 than during cropping year 2011–12 (Fig. 3), and on the whole approximately 30% more CO_2_ emissions were recorded during cropping year 2010–11 than during 2011–12 (Fig. 3).

CO_2_ emissions varied for all of the tillage methods and N fertilizer levels. For the chisel plow tillage treatment, using 160 kg N/ha reduced the emissions of CO_2_ (data not shown) and the CO_2_ emissions trend in case of the chisel plow tillage method and different N fertilizer levels was N_4_>N_3_>N_0_>N_1_>N_2_ (data not shown) (Table 1). For the Zero tillage planting method and N fertilizer level interactions, the CO_2_ emission varied but the emissions trend was N_0_>N_3_>N_2_>N_1_>N_4_ (data not shown) (Table 1). For the rotary tillage planting method and N fertilizer level interactions, the CO_2_ emissions trend was N_4_>N_3_>N_1_>N_0_>N_2_ (data not shown) (Table 1). Similarly for the mold board plow tillage method and N fertilizer level interactions, the CO_2_ emissions trend was N_0_>N_4_>N_1_>N_3_>N_2_ (data not shown) (Table 1).

**Table 1:** ANOVA (Mean Square Values) of biological yields, grain yields, soil organic carbon, and cumulative emissions of CO_2_ during two cropping years (2010–12).

Source	D.F	B.Y	G.Y	SOC	CO_2_
Tillage methods	3	6593271.9***	122260.74**	32.12145866***	9605737865***
Planting years	1	13496603.4***	29504910.64***	97.90534734***	8211670776***
Mulch kinds	1	132977.5NS	20786.14NS	3.74523078***	93546720 NS
N Fertilizer levels	4	1470235.5***	4549170.27***	3.78932599***	21321766841***
Tillage methods X Mulch kinds	3	6780289.1***	1690508.29***	26.55366456***	1455896530***
Tillage methods X N Fertilizer levels	12	1443071.8 NS	302485.89NS	5.42559025***	1418530119***
Tillage methods X planting years	3	3443481.8NS	1276931.92*	12.85179871***	1455478300***
Planting years X Mulch kinds	1	66692.7NS	418751.64NS	0.67995212 NS	2547564904***
Planting years X N Fertilizer levels	4	10758689.1***	3983648.62***	3.45638215***	720140735***
Mulch kinds X N Fertilizer levels	4	975872.1NS	461889.16NS	3.23681715***	3518560905***
Tillage methods X Planting years X Mulch kinds	3	1162751.4NS	190004.99NS	12.61344035***	406313591***
Tillage methods X Planting years X N fertilizer levels	12	12108232.1NS	369168.67NS	4.50848299***	959919429***
Tillage methods X Mulch kinds X N fertilizer levels	12	1785389.2NS	420989.67NS	6.20244131***	2881830882***
Tillage methods X Mulch kinds X Planting years X N fertilizer levels	16	2029198.1 NS	782603.91***	4.05394235***	1326809061***

* Significant at 0.05 probability levels.

** Significant at 0.01 probability levels.

*** Significant at 0.001 probability levels.

B.Y, Biological yields., G.Y, Grain yields., SOC, Soil organic carbon.

X, indicates interactions between different factors i.e. Tillage's X Mulches indicates interactions between different tillage methods and mulch kinds.

With the exception of the mold board plow tillage method, corn residue mulch applications increased the CO_2_ emissions in all of the other three tillage methods. Lower CO_2_ emissions were recorded for all the tillage methods during cropping year 2011–12 in comparison to cropping year 2010–11 and throughout cropping year 2010–11, corn residue mulch applications increased the CO_2_ emissions to 16.5% compared to the non residue mulched treatments. Similarly during cropping year, 2011–12, 12.6% fewer CO_2_ emissions were recorded for the corn residue mulched treatments in comparison to the non-residue mulched treatments (data not shown) (Table 1).

For the N_0_ (0 kg N/ha), N_1_ (80 kg N/ha), N_2_ (160 kg N/ha) and N_4_ (320 kg N/ha), treatments, the use of corn residue mulch increased the CO_2_ emissions by approximately 6.2%, 5.5%, 0.6% and 35.6%, respectively, and for N_4_ (240 kg N/ha), the application of corn residue mulch reduced the CO_2_ emissions by approximately 35.6% (data not shown) (Table 1).

### Soil temperatures versus CO_2_ emissions

**Figure 4:**
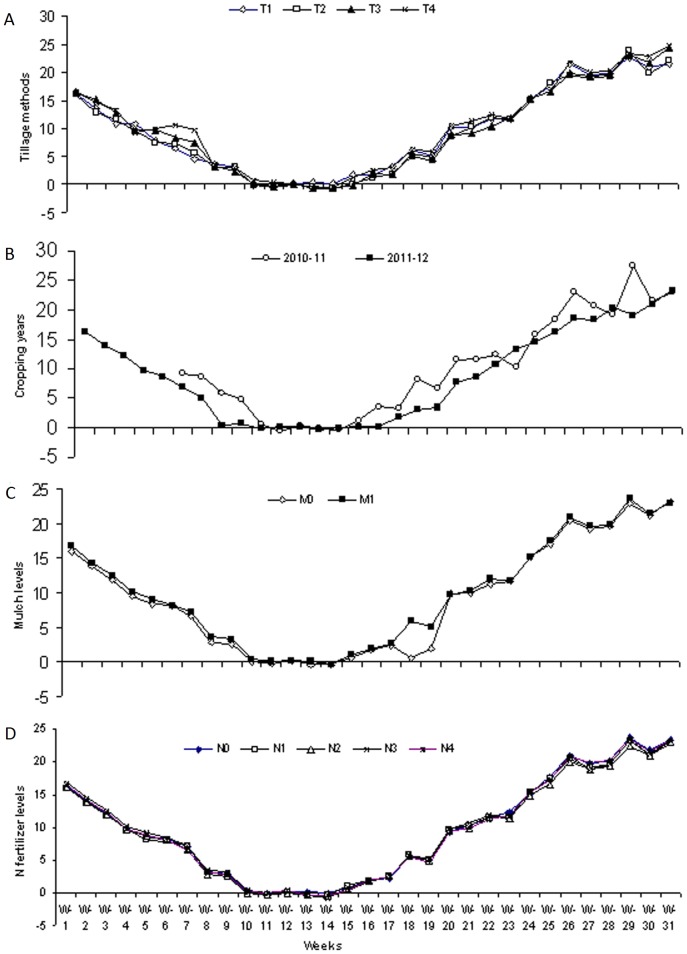
Changes in soil temperatures (0–5 cm depth) due to different tillage methods, corn residue mulch and N fertilizer levels during two cropping years (2010–12). (A). Changes in soil temperatures (0–5 cm depth) due to different tillage methods i.e. T_1_, chisel plow tillage, T_2_, zero tillage, T_3_, rotary tillage and T_4_, mold board plow tillage method., (B) Changes in soil temperatures (0–5 cm depth), as affected by different tillage methods, different mulch kinds and different N fertilizer levels during two cropping years (2010–12)., (C), Changes in soil temperatures (0–5 cm depth) during two cropping years (2010–12) due to different mulch kinds i.e. M_0_, no mulch and M_1_, corn residue mulch., (D). Changes in soil temperatures (0–5 cm depth) due to different N fertilizer levels during two cropping years (2010–12) including, N_0_, 0 kg N/ha., N_1_, 80 kg N/ha., N_2_, 160 kg N/ha., N_3_, 240 kg N/ha and N_4_, 320 kg N/ha.

Temperature changes for the top 5 cm of soil from the different treatments during the two years (2010–12) of study are given in Fig. 4. Although with the passage of time, there were variations in soil temperatures for the different tillage methods, however intermediary types of temperature changes were recorded following zero tillage planting in comparison to the other three tillage methods (Fig. 4). Similarly, although statistically comparable temperatures were recorded for both types of mulch treatments, slightly increased temperatures were recorded in the corn residue mulched treatments relative to the non-residue mulched treatments (Fig. 4). However, no soil temperature differences were recorded for the different N fertilizer level treatments (Fig. 4). Generally speaking, higher soil temperatures were recorded during the cropping year 2010–11 than during cropping year 2011–12 (Fig. 4). CO_2_ emission trends showed that CO_2_ emissions increased with an increase in soil temperatures and vice versa (Figs. 2, 4).

### Soil moisture versus CO_2_ emissions

**Figure 5:**
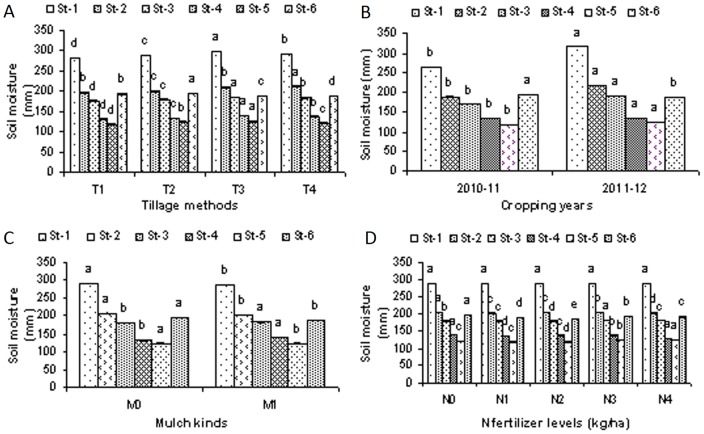
Total soil moisture contents (0–100 cm soil depth) as affected by different tillage methods, mulch kinds, and N fertilizer levels during two cropping years (2010–12). (A). Total soil moisture contents (0–100 cm soil depth) due to different tillage methods i.e. T_1_, chisel plow tillage., T_2_, zero tillage., T_3_, rotary tillage and T_4_, mold board plow tillage method., (B).Total soil moisture contents (0–100 cm soil depth) as affected by different tillage methods, different mulch kinds and different N fertilizer levels, during two cropping years (2010–12).(C), Total soil moisture contents (0–100 cm soil depth) as affected by different mulch kinds during the two cropping years (2010–12) i.e. M_0_, no mulch and M_1_, corn residue mulch., (D). Total soil moisture contents (0–100 cm soil depth) as affected by different N fertilizer levels, during two cropping years (2010–12) including, N_0_, 0 kg N/ha., N_1_, 80 kg N/ha, N_2_, 160 kg N/ha., N_3_, 240 kg N/ha and N_4_, 320 kg N/ha., (*) Stage-1, (Crop sowing stage), Stage-2, (Crop revival stage), Stage-3, (Stem elongation stage), Stage-4, (Booting stage), Stage-5, (Grain formation stage) and Stage-6 (Crop harvesting stage).

When compared to all of the other tillage methods, the highest soil water contents were recorded in response to the rotary tillage planting method (Fig. 5). Similarly, higher water contents were recorded during the cropping year 2011–12 for the different crop growth stages relative to cropping year 2010–11 (Fig. 5). Corn residue mulch increased the soil moisture contents of the crop revival stage and on the booting stage compared to the non-residue mulched treatments (Fig. 5). Lower soil moisture contents were recorded for the 80 kg N/ha (N_1_) and 160 kg N/ha (N_2_), treatments compared to all of the other N fertilizer level treatments (Fig. 5). Two years of mean data show that the CO_2_ emissions were lower in those tillage methods or N fertilizer levels treatments that had lower water contents (Fig. 2; Fig. 4).

### Soil organic carbon versus CO_2_ emissions

**Figure 6:**
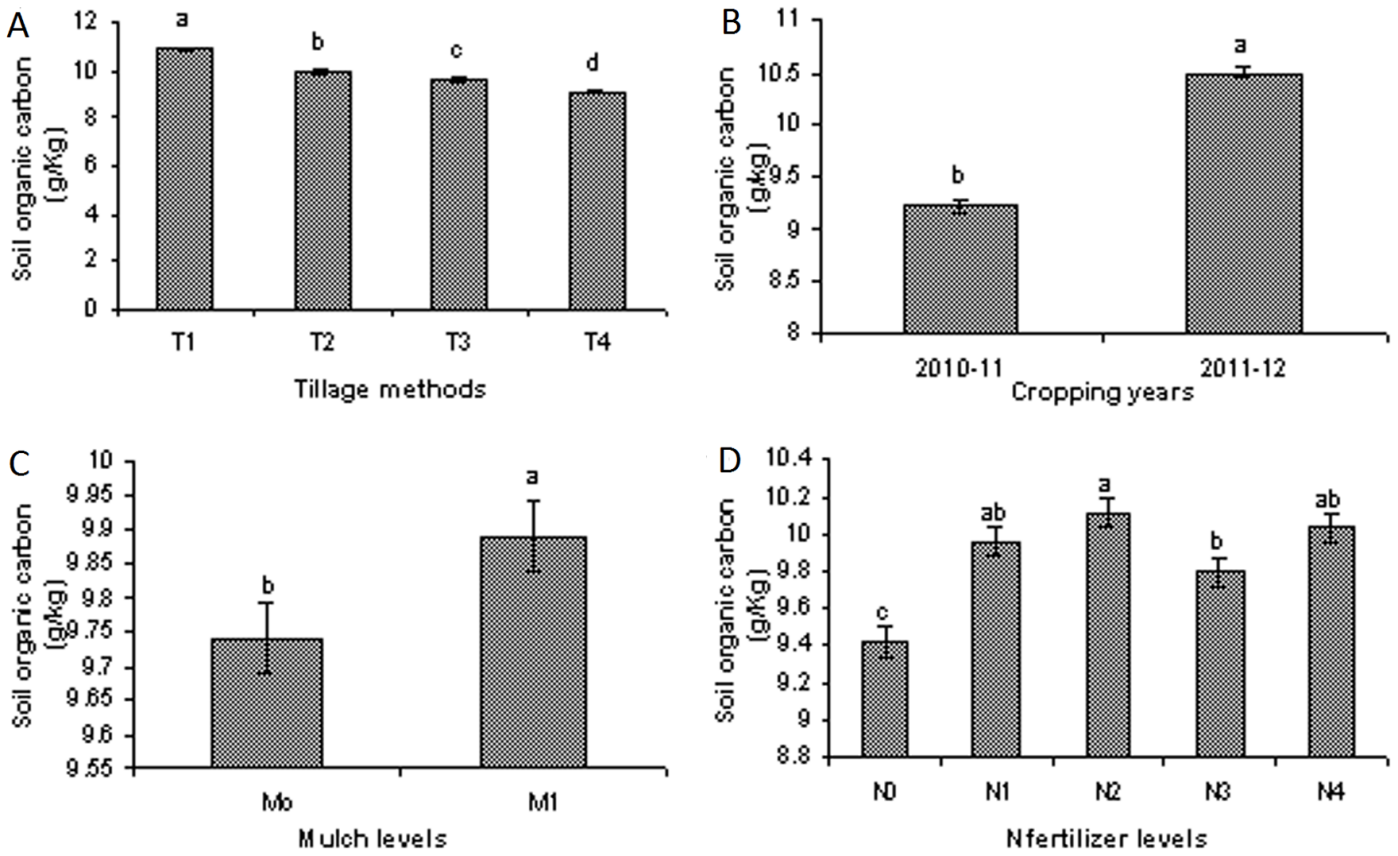
Soil organic carbon (SOC) contents (g/kg) as affected by different tillage methods, different mulch kinds and different N fertilizer levels during different cropping years (2010–12). (A), Soil organic carbon (SOC) contents as affected by different tillage methods i.e. T_1_, chisel plow tillage., T_2_, zero tillage., T_3_, rotary tillage and T_4_, mold board plow tillage method., (B), Mean soil organic carbon (SOC) contents as affected by different tillage methods, mulch kinds and different N fertilizer levels during two cropping years (2010–12)., (C), Soil organic carbon (SOC) contents as affected by different mulch kinds i.e. M_0_, no corn residue mulch and M_1_, corn residue mulch., (D), Soil organic carbon (SOC) contents as affected by different N fertilizer levels during two cropping years (2010–12) including, N_0_, 0 kg N/ha., N_1_, 80 kg N/ha., N_2_, 160 kg N/ha., N_3_, 240 kg N/ha and N_4_, 320 kg N/ha.

Two years of mean data show that the tillage methods had significant effects on the SOC from 0–10 cm soil depths and that compared to all of the tillage methods, the highest SOC was recorded following chisel Plow tillage (Fig. 6). Higher SOC contents were recorded for all of treatments during cropping year 2011–12 in comparison to cropping year 2010–11 (Fig. 6). The use of corn residue mulch increased the SOC compared to the non-mulched treatments (Fig. 6), and higher over all SOC contents were recorded in the cases of N_1_ (80 kg N/ha) and N_2_ (160 kg N/ha) in comparison to all of the other N fertilizer levels treatments (Fig. 6). The data show that CO_2_ emissions were lower in those treatments that had higher SOC contents (Fig. 3; Fig. 6).

### Effects of seasonal variations on CO_2_ emissions

**Figure 7:**
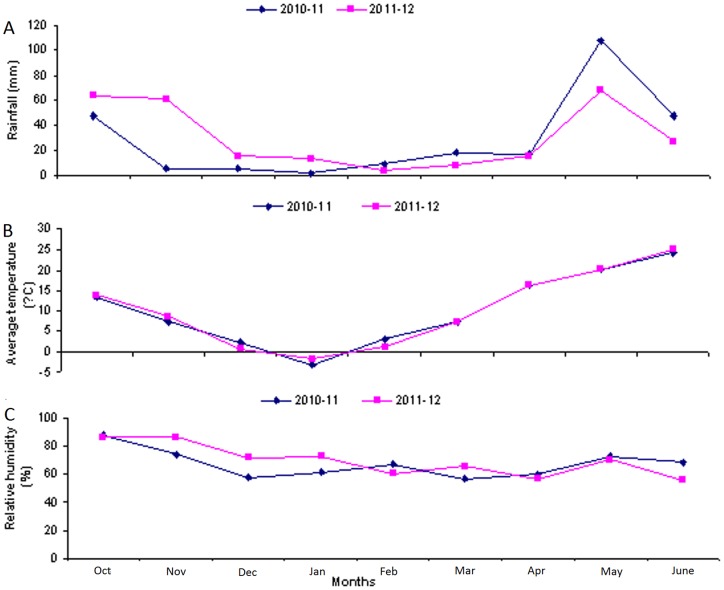
Monthly rainfalls, average temperatures and average relative humidity's of the study area during two wheat crop growing seasons (2010–12). (A).Rainfalls of the study area during two wheat crop growing seasons (2010–12), (B) Average temperatures of the study area during two wheat crop growing seasons (2010–12) (C) Average relative humidities of the study area during two wheat crop growing seasons (2010–12). (*). R.F-1, Rainfalls during cropping year 2010–11., R.F-2, rainfalls during cropping year 2011–12., Av.temp-1, Monthly average temperatures during the cropping year 2010–11., Av.temp-2, Monthly average temperatures during the cropping year 2011–12., R.H (%)-1, Average monthly relative humidities during the cropping year 2010–11., R.H (%)-2, Average monthly relative humidities during the cropping year 2011–12.

**Figure 8:**
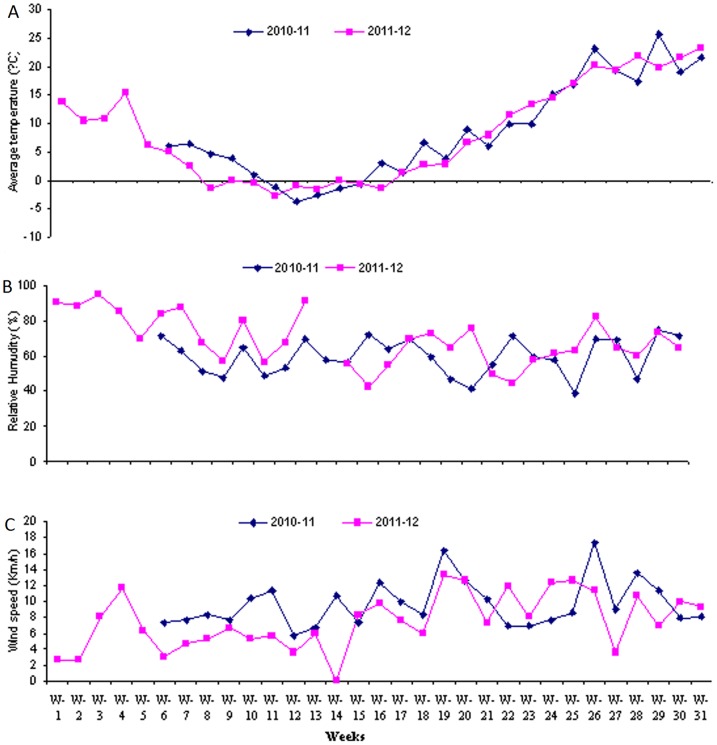
Average weekly temperatures, relative humidity's and wind speeds during the two wheat crop growing seasons (2010–12). (A) Average weekly temperatures of the study area during the two wheat crop growing seasons (2010–12), (B).Average weekly relative humidity's of the study area during the two wheat crop growing seasons (2010–12), (C) Average weekly wind speeds of the study area during the two wheat crop growing seasons (2010–12).

Seasonal temperature variations had significant effects on the CO_2_ emissions. Because there were normal temperatures when the wheat crops were sown, higher CO_2_ emissions were recorded, but reduced emissions were recorded with the decline in temperature during the winter seasons. With increased of crop growth and ascending of seasonal temperatures higher CO_2_ emissions were recorded during both years (Figs. 2, 3, 4, 7, 8,).

## Discussion

### Soil CO_2_ fluxes

Variations in seasonal temperatures had significant effects on soil temperatures, which ultimately affected the CO_2_ emissions (Figs. 2, 3, 4, 7, 8). Higher CO_2_ fluxes were recorded immediately after the tillage operations, which continued for a few days, and these emissions decreased with the passage of time (Fig. 2). Our results are in agreement with other findings [35]. Other investigators have reported changes in CO_2_ emissions with seasonal variations. According to these investigators, seasonal variations in CO_2_ emissions are controlled not only by the soil temperatures and soil moistures but also by the tillage practices. Changes in CO_2_ emissions from seasonal variations have been reported for almost all ecosystems. These emissions mainly depend both on the type of climate and the ecosystem [36].

Similar findings regarding variations in CO_2_ fluxes with changes in soil temperatures and crop growth stages have been reported for rice crops [37]. In our study, the air and soil temperatures had significant effects on CO_2_ emissions. Thus with the decrease in soil temperatures during the winter months, the CO_2_ emissions decreased, and the CO_2_ emissions again increased with the rise in soil temperatures during the summer months (Figs. 2, 3, 4, 7, 8).

Generally, the seasonal CO_2_ emissions variations found in our experiment were similar to other findings [38]. Other investigators reported that these emission variations might be related to variations in autotrophic and in heterotrophic respiration because both are involved in soil CO_2_ emissions. In addition a large amount of CO_2_ is released from plant roots, during the continuation of the plant-energy system. Microbial and root respiration can also significantly contribute to CO_2_ emissions.

### Tillage method effects on CO_2_ emissions

Higher CO_2_ emissions were recorded immediately after the tillage operations, which continued for a few days (Fig. 2). Our results are in agreement with findings from other researchers, who reported that CO_2_ emissions following tillage increased up to 2–15 times [39], [40], [41], [42] and [43]. According to these findings [39], instead of microbial activity, the basic reason for higher CO_2_ emissions immediately after the tillage was actually the release of entrapped CO_2_ from the soil pores as a result of physical operations. The other reasons for these higher emissions might be that (1) tillage operations break soil aggregates and expose their organic matter to microbial attack [44], [45]; (2) tillage operations encourage the mineralization of soil organic matter by incorporating crops residues into the soil [46]; and (3) tillage operations enhance soil aeration [43]. In our study, the tillage methods had significant effects on the CO_2_ emissions and, overall, the rotary tillage and mold board plow tillage methods led to higher CO_2_ emissions compared to the chisel plow and zero tillage planting methods (Fig. 2 and Fig. 3). Similar findings under different tillage systems have been previously reported [13]. These researchers reported significantly more CO_2_ emission fluxes from the fields tilled by a mold board plow relative to the fields prepared by the chisel plow methods. According to these researchers, the basic reason for the greater emission fluxes from the mold board plow tillage method compared to the chisel plow tillage method was the depth and extent to which the soil was disturbed by using the different tillage implements. In our experiment, higher soil temperatures in the top 5 cm depth (Fig. 4) and generally higher moisture contents (Fig. 5) in the rotary tillage and mold board plow methods might be responsible for the higher emissions, in addition to the soil preparation depths. The tillage depths also resulted in a reduction of the SOC in the top 0–10 cm soil layers for the rotary tillage and mold board plow methods, compared to the chisel plow tillage and zero tillage planting methods (Fig. 6).

### Corn residue mulch effects on CO_2_ emissions

Weekly CO_2_ emissions data (Figs. 2, 4) show that during cropping year 2010–11, the application of corn residue mulch caused an overall increase in CO_2_ emissions compared to the non-residue mulched treatments, but during the cropping year 2011–12, fewer CO_2_ emissions were recorded in response to the application of corn residue mulch relative to the non-residue mulched treatments (Fig. 2 and Fig. 4). Although more CO_2_ emissions fluxes were recorded in the corn residue mulched treatments in comparison to the non-residue mulched treatments, these might be due to the more microbial activities in the corn residue mulched treatments, which might have increased the SOC with the passage of time (Fig. 6). However, the two year mean data show that statistically non significant differences were recorded for CO_2_ emissions following the use of the corn residue mulched or non mulched treatments. These findings might be explained by noting that both years CO_2_ collecting chambers were fixed before the application of corn residue mulch. As a result there were fewer corn residues within the CO_2_ collecting chambers from the corn residue mulched treatments. This smaller amount might be the reason why no CO_2_ emission differences were recorded following the applications of different corn residue mulch treatments. The other reason might be that during the cropping year 2010–11, the application of corn residue mulch increased the CO_2_ emissions but this mulch also increased the SOC contents of the corn residue mulched treatments, possibly resulting in the lower emission of CO_2_ from the corn residue mulched treatments during the year 2011–12 (Fig. 3). A modeling study reported [47] that instead of applying higher rates of fertilizers, the use of crop residues or manure amendments would mitigate GHG emissions more efficiently. Similarly, it has been reported [48] that applications of straw increased the SOC sequestration in the soil which ultimately influenced the temporal patterns of CO_2_ emissions from the soil.

### N fertilizer level effects on CO_2_ emissions

Very few studies regarding CO_2_ emissions in relation to the different tillage methods, corn residue mulch and N fertilizer levels have previously been reported in this region of China. It is expected that the application of inorganic N fertilizers along with organic materials will affect the mineralization of soil organic matter and crop productions, which will ultimately affect CO_2_ emissions [8].

However variations in CO_2_ emissions following fertilizer applications have been reported for different areas of China. Some scientists [15] have reported that the fertilizer applications suppresses CO_2_ emissions and others [49] have reported that fertilizer application enhances CO_2_ emissions. Moreover, some other scientists [19] have reported that fertilizer applications have no effects on CO_2_ emissions.

Our study shows that the use of 80 kg and 160 kg N/ha, suppressed CO_2_ emissions when compared with the 0 N fertilizer level, but that further increases in N fertilizer application rates enhanced CO_2_ emissions. Higher emissions from the N_0,_ nitrogen fertilizer treatments might be explained by noting that plants under unfertilized N treatments are considered to respond to a relative shortage of N by increasing the plant's carbon allocation to its structures and functions, which are responsible for N acquisition [50]. In our study, the use of different levels of N fertilizer relative to the no nitrogen fertilizer level significantly increased crop yields (Fig. 1).This result shows that the higher CO_2_ emission fluxes in response to higher levels of N fertilizer might be explained by the increased use of C for microbial growth [51] and it might also be explained by the less efficient use of carbon by the microbial biomass, which resulted in a greater proportion of carbon loss in the form of CO_2_ fluxes [52].

In our case, the use of corn residue mulch in combination with different levels of N fertilizer increased CO_2_ emissions following the chisel plow, zero tillage and rotary tillage planting methods (Table 1). Our results regarding CO_2_ emissions following the combination of N fertilizer and organic amendments are in agreement with the results of other scientists [53], [38] and [54]. These investigators reported a higher CO_2_ emission flux from the treatments that utilized both fertilizers and organic manures. This finding shows that up to a certain extent the use of higher N fertilizer levels suppresses CO_2_ emissions but in our case, higher levels of N fertilizer (i.e., 240 and 320 kg N/ha) enhanced CO_2_ emissions. These results are contrary to the findings of other scientists [30] and [31]. These investigators reported no clear reasons for the CO_2_ emission reductions.

### Cropping year effects on CO_2_ emissions

Two years of data on a weekly as well as on a cumulative basis show that there were more CO_2_ emissions from all of the treatments during cropping year 2010–11 compared to the CO_2_ emissions during cropping year 2011–12 (Figs. 2, 3). The main reasons for these lower emissions during cropping year 2011–12 were, on the whole, lower air temperatures, higher relative humidities and higher over all rainfall concentrations during the early periods of wheat crop growth relative to cropping year 2010–11 (Fig. 7). These factors all ultimately reduced the soil temperatures, which resulted in reduced CO_2_ emissions during the cropping year 2011–12 compared to cropping year 2010–11 (Figs. 4, 7, 8). Another reason for the lower emissions might be an increase in the SOC from the corn residue mulched treatments, which might have ultimately helped to reduce the CO_2_ emissions during cropping year 2011–12 in comparison to cropping year 2010–11 (Figs. 2, 3, 6).

### Effects of soil temperatures, soil moisture and soil organic carbon on CO_2_ emissions

Respiration of ecosystem mainly depends on both the heterotrophic (microbe) and autotrophic (plant) activities and both of these factors are controlled by the prevailing environmental conditions (basically temperature and water availability), availability of carbohydrates and substrates and others [55], [56] and [57]. Many studies have shown that seasonal variations in CO_2_ emissions were mainly caused by the soil temperature, soil moisture or the combination of both these factors [58], [36].

Our study also indicates that soil temperature was an important driving force for the increased CO_2_ emissions, which is also supported by the Q10 values given in Table 2 and Table 3. It has also been reported that the CO_2_ evolution rate significantly increases with an increase in temperature and moisture [37]. Our results are also in agreement with many other field studies, which have shown strong relationships between soil temperatures and CO_2_ flux rates [59], [60], [17] and [18]. Additionally, our results agree with the findings of [61], who reported a stronger polynomial for temperature and moisture interaction (r^2^ = 0.89) than for temperature alone (r^2^ = 0.47). Many previous studies have reported that changes in crop management practices, i.e., the appropriate use of tillage operations, proper fertilization, crop residue applications and crop rotations can be helpful for managing soil organic matter, e.g.,. [62].

**Table 2:** Q10 values of the different treatments during the different cropping years (2010–12).

Treatments	chisel plow		zero tillage		rotary tillage		mold board plow tillage	
	2010–11	2011–12	2010–11	2011–12	2010–11	2011–12	2010–11	2011–12
	Y = 69.220e^0.0516x^	Y = 47.81 e^0.0898x^	Y = 73.371e^0.0736x^	Y = 74.836e^0.807x^	Y = 140.49e^0.0427x^	Y = 67.628 ^0.0749x^	Y = 287.91e^0.0707x^	Y = 78.941e^0.1001x^
N_0_	R^2^ = 0.7076	R^2^ = 0.8064	R^2^ = 0.0823	R^2^ = 0.7213	R^2^ = 0.7472	R^2^ = 0.7372	R^2^ = 0.6076	R^2^ = 0.7439
	Q10 = 1.68	Q10 = 2.43	Q10 = 2.09	Q10 = 1.90	Q10 = 2.54	Q10 = 2.55	Y = 287.91e^0.0707x^	Q10 = 2.72
	Y = 79.76e^0.0744x^	Y = 73.979e^0.0816x^	Y = 57.341e^0.0845x^	Y = 62.738e^0.0996x^	Y = 142.39e^0.0691x^	Y = 93.744e^0.092x^	Y = 247.36e^0.0385x^	Y = 129.37e^0.0818x^
N_1_	R^2^ = 0.6672	R^2^ = 0.7158	R^2^ = 0.0869	R^2^ = 0.752	R^2^ = 0.8187	R^2^ = 0.8074	R^2^ = 0.5688	R^2^ = 0.736
	Q10 = 2.10	Q10 = 2.26	Q10 = 2.33	Q10 = 2.71	Q10 = 1.63	Q10 = 2.11	Q10 = 2.03	Q10 = 2.72
	Y = 80.442e^0.0827x^	Y = 0.787e^0.1012x^	Y = 114.12e^0.0509x^	Y = 57.382e^0.0891x^	Y = 90.813e^0.0616x^	Y = 122.76e^0.0669x^	Y = 108.97e^0.0634x^	Y = 79.756e^0.0873x^
N_2_	R^2^ = 0.5944	R^2^ = 0.8201	R^2^ = 0.528	R^2^ = 0.7701	R^2^ = 0.790	R^2^ = 0.6534	R^2^ = 0.8134	R^2^ = 0.7563
	Q10 = 2.29	Q10 = 2.75	Q10 = 1.66	Q10 = 2.70	Q10 = 2.00	Q10 = 2.51	Q10 = 1.47	Q10 = 2.27
	Y = 125.25e^0.1085x^	Y = 70.222e^0.0931x^	Y = 82.409e^0.0478x^	Y = 55.391e^0.1006x^	Y = 176.74e^0.097x^	Y = 149.95e^0.0859x^	Y = 103.92e^0.0614x^	Y = 69.581e^0.0843x^
N_3_	R^2^ = 0.7977	R^2^ = 0.7054	R^2^ = 0.7439	R^2^ = 0.7937	R^2^ = 0.6429	R^2^ = 0.7876	R^2^ = 0.852	R^2^ = 0.6457
	Q10 = 2.96	Q10 = 2.47	Q10 = 2.33	Q10 = 2.02	Q10 = 1.85	Q10 = 1.95	Q10 = 1.89	Q10 = 2.32
	Y = 55.139e^0.0653x^	Y = 61.6e ^0.0904x^	Y = 43.884e^0.0903x^	Y = 60.312e^0.8051x^	Y = 90.419e^0.058x^	Y = 221.26e^0.0624x^	Y = 116.85e^0.0613x^	Y = 96.28e^0.1086x^
N_4_	R^2^ = 0.6443	R^2^ = 0.7961	R^2^ = 0.7996	R^2^ = 0.9015	R^2^ = 0.6951	R^2^ = 0.6654	R^2^ = 0.7312	R^2^ = 0.7969
	Q10 = 1.02	Q10 = 2.18	Q10 = 1.61	Q10 = 2.16	Q10 = 2.64	Q10 = 2.32	Q10 = 1.85	Q10 = 2.96

N_0_, 0 kg N/ha., N_1_, 80 kg N/ha., N_2_, 160 kg N/ha., N_3_, 240 kg N/ha and N_4_, 320 kg N/ha.

**Table 3:** Q10 values of the different treatments during the different cropping years (2010–12).

Treatments	chisel plow		zero tillage		rotary tillage		mold board plow tillage	
	2010–11	2011–12	2010–11	2011–12	2010–11	2011–12	2010–11	2011–12
	Y = 43.137e^0.1316x^	Y = 87.49e^0.0778x^	Y = 163.72e^0.0363x^	Y = 61.295e^0.09x^	Y = 62.946e^0.1111x^	Y = 99.78e^0.0961x^	Y = 183.66e^0.0518x^	Y = 55.625e^0.0947x^
N_0_+M	R^2^ = 0.8849	R^2^ = 0.6632	R^2^ = 0.4067	R^2^ = 0.8372	R^2^ = 0.7113	R^2^ = 0.8096	R^2^ = 0.753	R^2^ = 0.8216
	Q10 = 3.72	Q10 = 2.69	Q10 = 2.47	Q10 = 2.10	Q10 = 1.79	Q10 = 2.96	Q10 = 1.85	Q10 = 2.58
	Y = 254.21e^0.0484x^	Y = 0.0988e^0.0516x^	Y = 84.882e^0.0413x^	Y = 49.272e^0.0965x^	Y = 175.25e^0.0557x^	Y = 76.273e^0.0858x^	Y = 112.84e^0.0703x^	Y = 51.454e^0.0957x^
N_1_+M	R^2^ = 0.7689	R^2^ = 0.8367	R^2^ = 0.7109	R^2^ = 0.7881	R^2^ = 0.5183	R^2^ = 0.8149	R^2^ = 0.695	R^2^ = 0.7376
	Q10 = 1.62	Q10 = 2.52	Q10 = 1.44	Q10 = 2.22	Q10 = 3.04	Q10 = 2.58	Q10 = 1.68	Q10 = 2.60
	Y = 72.653 ^0.0809x^	Y = 45.736e^0.0926x^	Y = 80.339e^0.0722x^	Y = 61.262e^0.0907x^	Y = 163.02e^0.0519x^	Y = 104.06e^0.083x^	Y = 93.668e^0.0666x^	Y = 46.012e^0.1166x^
N_2_+M	R^2^ = 0.8237	R^2^ = 0.7309	R^2^ = 0.8477	R^2^ = 0.7992	R^2^ = 0.4896	R^2^ = 0.7173	R^2^ = 0.6503	R^2^ = 0.816
	Q10 = 2.25	Q10 = 2.87	Q10 = 1.52	Q10 = 2.18	Q10 = 1.95	Q10 = 2.36	Q10 = 2.02	Q10 = 3.17
	Y = 1.015e^0.0898x^	Y = 46.994e^0.1054x^	Y = 164.85e^0.0502x^	Y = 30.192e^0.1197x^	Y = 163.9e^0.405x^	Y = 40.179e^0.0983x^	Y = 75.02e^0.0742x^	Y = 72.535e^0.1075x^
N_3_+M	R^2^ = 0.8604	R^2^ = 0.8214	R^2^ = 0.4050	R^2^ = 0.8784	R^2^ = 0.6464	R^2^ = 0.84	R^2^ = 0.7363	R^2^ = 0.8221
	Q10 = 2.45	Q10 = 1.11	Q10 = 2.06	Q10 = 2.18	Q10 = 1.68	Q10 = 2.29	Q10 = 1.95	Q10 = 2.93
	Y = 8.69e^0.10179x^	Y = 69.932e^0.0859x^	Y = 51.822e^0.0641x^	Y = 36.672e^0.1073x^	Y = 287.97e^0.0937x^	Y = 121e^0.1050x^	Y = 127.88e^0.0702x^	Y = 73.455e^0.1177x^
N_4_+M	R^2^ = 0.8342	R^2^ = 0.7264	R^2^ = 0.7247	R^2^ = 0.8364	R^2^ = 0.6465	R^2^ = 0.854	R^2^ = 0.7656	R^2^ = 0.8821
	Q10 = 2.76	Q10 = 2.36	Q10 = 1.65	Q10 = 2.74	Q10 = 1.50	Q10 = 2.67	Q10 = 2.10	Q10 = 3.24

N_0_+M_,_ 0 kg N/ha + corn residue mulch., N_1_+M, 80 kg N/ha + corn residue mulch., N_2_+M, 160 kg N/ha + corn residue mulch., N_3_+M, 240 kg N/ha + corn residue mulch., N_4_+M, 320 kg N/ha + corn residue mulch.

Our study also shows that the results at the end of cropping year 2010–11 revealed more SOC in the top 0–10 cm of soil than that at the end of cropping year 2011–12. This result shows that the adaptation of different management practices, i.e., the application of crop residues, increased the SOC contents, especially following chisel plow tillage and zero tillage planting compared with rotary tillage and mold board plow tillage. SOC sequestration is a long term processes and various results have been previously reported, i.e. [63] and [64], have reported that conservation tillage is a recommended management practice for agricultural ecosystems that can enhance the pool of soil organic carbon (SOC), in the soil. Following an analysis of global data, NT reportedly sequestered carbon at an average rate of 0.57 Mg C ha^−1^ compared with the mold board plow [65]. It has also been reported that increases in the SOC pools can be credited to either reductions in the CO_2_ efflux from the soil or to increases in the C inputs [65]. When comparing the soil surface across different tillage systems, conservation tillage systems retain more crop residues, which ultimately result in the formation of more SOC [66] and [67]. In addition to this finding, the decomposition process of surface applied plants residues as a part of conservation tillage is slow compared to conventional tillage systems because of lower contact with the soil.

In our study a negative but highly significant correlation coefficient (r) value i.e. −0.19403** was recorded between the SOC and cumulative CO_2_ emission. This finding might help in the reduction of CO_2_ emissions from the chisel plow tillage and zero tillage planting methods compared to the rotary tillage and mold board plow methods (Figs. 2 and 3). In our experiment, increases in the SOC contents from different tillage methods, especially chisel plow tillage and zero tillage, might be related to the lower disturbance of the soil, retention of more crop residues on the soil surface and reductions in the efflux of CO_2_ from the soil. Similar results have been reported by [65], [66] and [67].

## Conclusions

Intensive tillage and higher N fertilization are not only detrimental to the soil but are also destructive for the entire environment. Adaptations of appropriate tillage methods, crop residue applications and proper fertilization are beneficial for the soil as well as for the environment. These practices are also economically beneficial for resource-poor farmers. The findings from our study clearly indicate that the tillage methods significantly affected CO_2_ emissions and the zero tillage planting method emitted the lowest CO_2_ compared with the other three tillage methods. No significant differences in CO_2_ emissions were recorded for the applications of corn residue mulch, but the applications of corn mulch significantly improved the soil organic carbon (SOC) contents of the soil for all of the tillage systems. In addition, corn residue mulch application reduced the weed infestation by up to 40% (data not shown). Therefore, the application of corn residue mulch can be helpful for reducing the use of herbicides, which will also be helpful in establishing a healthy environment. Applications of different N fertilizer levels also significantly affected CO_2_ emissions and, overall, the lowest emissions were recorded for the 160 kg N/ha treatment. Higher CO_2_ emissions were recorded immediately after the tillage operations. This study also indicated that both soil temperatures and moistures strongly affected CO_2_ emissions and that compared with the other tillage methods, zero tillage planting gave better grain yields. The lowest N fertilizer use gave equal yields, for the two year mean, as did the application of higher N fertilizer levels. These results clearly indicate that proper changes in farm management practices i.e., the adoption of zero tillage, crops residue application and optimum use of N fertilizers can reduce CO_2_ emissions from soils. Therefore this type of long term study can be further helpful in reducing the emissions of CO_2_ from soils, which will be helpful in reducing the use of inorganic fertilizers. These practices will be helpful in reducing production costs and will be beneficial for the entire environment.

## Materials and Methods

### Experimental site

A two-year (2010–12)), field study was conducted at the experimental area of Northwest A&F University, Shaanxi Province, northwestern China (latitude of 34°20° N, longitude of 108° 04°E and elevation of 466.7 m above sea level) on the Eum-Orthrosols (Chinese soil Taxonomy) soil, with a mean bulk density of approximately 1.29 g cm^3^. The soil in the top 40 cm had an SOC of approximately 14.26 g/kg, total nitrogen 0.74 g/kg and the pH was approximately 7.85. This area is under the corn-wheat rotation system. During both years, the wheat crop was planted after harvesting the corn crop. Both fertilizers, i.e., phosphorous in the form of calcium phosphate (Ca_2_ (PO_4_)_3_ with 16% P and nitrogen in the form of urea with N≥46%, were applied to the corn crop at the rate of 750 kg/ha and 375 kg/ha, respectively. The total rainfall during the wheat crop growing season (October-June) was 231.6 mm and 242.7 mm during, cropping years 2010–11 and 2011–12 respectively (Fig. 7).

### Experimental design and treatments

A factorial experiment having a strip split-split arrangement, with tillage methods in the main plots, mulch levels in the sub plots and N-fertilizer levels in the sub-sub plots with three replicates, was used for this study. Different tillage methods, i.e., chisel plow tillage (T_1_), zero tillage (T_2_), rotary tillage (T_3_) and mold board plow tillage (T_4_) methods, were kept in the main plots, different mulch kinds, i.e., M_0_ (no residue mulch) and M_1_ (corn residue mulch), in the sub plots, while different nitrogen fertilizer rates, i.e., 0, 80, 160, 240 and 320 kg N/ha, were kept in the sub-sub plots. The area was uniform in terms of fertility. The total experimental plot area (3300 m^2^) was equally divided into four main tillage treatments. The area of each tillage treatment (33 m×25 m) was further sub-divided into sub–plots for mulch treatments, and finally the sub plots were further divided into sub-sub plots, and each sub-sub individual plot had an area of 3 m×25 m. Treatments were randomized within each sub-plot.

Chisel plow tillage (T_1_), was performed using a chisel plow. Following fertilizer applications, a chisel plow with a shank spacing of approximately 40 cm apart and 30–35 cm deep was used once. Later on, fertilizers were mixed by using the rotavator for up to a 5 cm depth. For zero tillage (T_2_) fertilizers were mixed by using the rotavator up to a 5 cm depth due to the lack of availability of a proper zero tillage drill. For rotary tillage (T_3_), the seed bed was prepared by using the rotavator up to a depth of 15–20 cm, and in the case of the mold board. plow tillage method (T_4_), the soil was plowed up to a 20–30 cm depth by using the mold board plow, followed by the rotavator for the final seed bed preparation.

Urea fertilizer with N≥46%, was used as the source of the nitrogen, and phosphorous (P) fertilizer in the form of calcium phosphate (Ca_2_ (PO_4_)_3_ with 16% P was equally applied to all of the treatments at a rate of 750 kg/ha at the time of soil preparation. The treatments arrangements were kept the same during both years (2010–12) of study. Previously harvested air-dried corn crops residues were used as the source of corn residue mulch during both years. When the wheat crop was at the 3–4 leaf stage, mulch was applied at a rate of 750 g/m^2^. The field was flat with a uniform topography. This area is rain fed, and a wheat-corn rotation is the main cropping system. No irrigation was applied to either crop. No changes were made to the areas with different tillage treatments, and a corn crop was planted after the wheat crop harvest by using the same tillage methods. The wheat crop was harvested using a combine harvester, and after harvesting the wheat crop, the corn crop was planted using a corn planter.

Winter wheat (C.V Shaan mai −139) was planted on October 17, 2010 and October 18, 2011 by using wheat drills. The line to line distance was maintained at approximately 16 cm apart. The seed had a 13% moisture contents and a 85% germination rate during both years. During cropping season 2010–11, a seed rate of about @ 190–200 kg/ha was used, while during cropping season 2011–12, a seed rate of approximately 205–210 kg/ha was employed. Experimental treatments were separated from each other by making boundaries between the treatments. Both years an herbicide application i.e. carfentrazone-ethyl (C_15_H_14_C_12_F_3_N_3_O_3_), was used to control the weeds. At physiological maturity, which occurred on June 8, 2011 and on June 10, 2012, three samples were randomly selected from each treatment and manually harvested by using the 1 m^2^ quadrants to calculate the grain yields. Finally the wheat crop was harvested using a combine harvester.

### Measurements

#### Meteorological factors

Meteorological data for the study area are given in Fig. 7 and Fig. 8, which indicate that during cropping year 2011–12, more rains were concentrated during the early period of crop growth relative to 2010–11, during which more rains were concentrated in the later crop growth stages. Higher average seasonal temperatures were recorded during the year 2010–11 than in 2011–12. However, more relative humidity was recorded during 2011–12 than in 2010–11. Weekly meteorological data showed higher, average temperatures during the year 2010–11 than during 2011–12. On the other hand, higher weekly relative humidities were recorded during the cropping year 2011–12 than during 2010–11. Similarly, higher weekly basis more wind speeds were recorded during the cropping year 2010–11 than during 2011–12.

#### Monitoring CO_2_ emissions

Because of the high number of treatments, both years of CO_2_ emissions data were recorded three times per week and one day of CO_2_ emissions data was used as one replicate for statistical analysis. CO_2_ emissions data were recorded using a GXH-3010E1 portable gas analyzer. This gas analyzer is made by the Beijing Huayun Yiqi Company, and the CO_2_ emission was recorded by using the method described by Gao et al [68]. During both years (2010–12) CO_2_ emissions data from all of treatments was recorded 6840 times, which included 3120 times during the cropping year 2010–11 and 3720 times during cropping year 2011–12.

One round PVC chamber (21 cm in diameter and 13.5 cm in height), having total area of approximately 0.0047 m^3^ was permanently fixed in the center of each treatment plot. The chamber was completely fixed in the soil up to a 4.5 cm depth. The plants growing with in the chambers were removed. As a consequence of some technical problems with the Gas analyzer, during wheat growing season 2010–11, the CO_2_ emissions data were recorded after the wheat had been planted for one month, and during cropping season, 2011–12, CO_2_ emissions were recorded starting during the first week of wheat planting. However, due to severe snow fall, data were not recorded on the 14^th^ week during this year. Taking data from wheat planting until harvest for 2010–11, the CO_2_ emissions data were recorded for 26 weeks, and during cropping year 2011–12, these data were recorded for 31 weeks. Every week, the data were collected 3 times depending upon the environmental condition, i.e., if the field was too wet from rainfall, then the data recording was stopped. Each time the data collection started at 900 a.m., and each sequence of CO_2_ flux measurements took at least 4 hours. Due to the large number of treatments the data were randomly collected from different treatments. The main purpose of this randomization was to minimize the effects of different days as well as changing soil temperatures on the emissions of CO_2_. The GXH-3010E1 gas analyzer was attached to the data collector chambers with intake and an outtake tubes. These tubes were made up of soft plastic pipes and each one was approximately one meter in length. At the time of data recording, first the CO_2_ data, i.e., X_1_ were recorded without closing/covering the chamber, and then the chamber top was tightly closed with a cover that had a fixed small fan. The gas within the chamber was mixed for three minutes with this fan. After this CO_2_ emission, data (X_2_) were recorded using the gas analyzer.

Chambers tops were closed for only three minutes at the time of data recording. To avoid any chemical change in the soil, these chambers were kept open for the whole remaining time. Along with CO_2_ data, soil temperatures data were also recorded from each treatment from depths of 5 cm. For this purpose, thermometers were permanently fixed in each plot each year for the whole crop growth period. The CO_2_ emission rate was calculated by using equation (1) as described by Gao et al. [68].
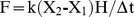
(1)Where F is the CO_2_ emission in mg/(m^2^.h); K is a constant with a value of 1.80 (25°C) and X_1_ and X_2_ are the CO_2_ emissions rates from the chambers before and after covering of the chambers. H is the height of the chambers in meters and 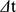 is the time in hours (h). The cumulative emission of CO_2_ was calculated using the following relationship, as described by Wilson H.M and Alkaisi W.W (2008) [69],
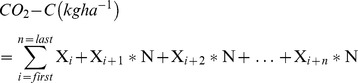
(2)where (i) is the first week of the growing season when the first CO2 emission rate was taken, (n) is the last week of the growing season when the last CO2 emission rate was taken, X is the CO2 rate (Kg ha−1 day−1), and N is the number of days between two consecutive CO2 emission rates measurements. Finally, these CO2 emission rates for the whole wheat crop growing period (taken between 9.00 A.M until approximately 1.00 P.M) were converted into tons/ha.

#### Soil moisture measurements

Both years soil moisture contents were measured during different crop growth stages, i.e., before planting the wheat crops, at the 5 leaf stage (Zadoks stage 1.0–1.9), stem elongation stage (Zadoks stage 3.0–3.9), booting stage (Zadoks stage 4.0–4.9), grain formation stage (Zadoks stage 7.0–7.9) and harvesting stage. For this purpose, soil samples were collected from each treatment from 0–100 cm soil depths with three replicates, with increments of 0–10 cm, 10–20 cm, 20–30 cm. 30–40 cm, 40–60 cm, 60–80 cm and 80–100 cm. Soil samples were collected in aluminum boxes using a hand auger, and fresh soil samples were immediately transported to the laboratory. After recording the fresh weights, these samples were dried in an oven at 105°C for at least 48 hours, and the soil water contents were then measured using equation (3) given below.
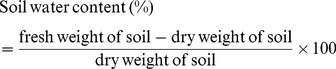
(3)


Soil water contents (mm) were calculated as gravimetric moisture contents using the equation (soil water ×B.D× thickness of soil layer). Soil bulk densities from the different tillage treatments and from the different soil depths were measured using the core method. Total soil water contents in the 0–100 cm soil depths of the different treatments were measured on the basis of the bulk densities of these different soil layers.

#### Soil organic carbon (SOC) measurements

Both year soil SOC samples were collected from the top 0–10 cm soil depth after the wheat harvest. Soil samples were collected from two randomly chosen points from each plot using a hand augur. The samples from each treatment were then mixed together to make a composite sample of each treatment. These samples were then air-dried at room temperature, crushed gently and passed through a 2 mm sieve for further chemical analysis. Soil organic carbon was determined by the oxidation method with K_2_ Cr_2_ O_7_ -H_2_SO_4_. For chemical analysis, 0.5 grams of soil was digested with 5 mL of 1 M K_2_ Cr_2_ O_7_ and 5 mL of concentrated H_2_SO_4_ and was heated at 175°C for 5 minutes followed by titration of the digests with FeSO_4_ [70].

### Statistics

Annual data collected for the CO_2_ emission rates and for other related parameters over the whole 2-year period were subjected to an analysis of variance (ANOVA) by using the factorial experiment with the strip-split-split arrangement having the tillage methods in the main plots, mulch in the sub plots and N-fertilizer levels in the sub-sub plots. The SAS analytical software package GLM (8.01) was used for the analyses. Mean values and standard errors (SE) were calculated for each treatment, and an ANOVA was used to assess the treatment effects on the measured variables. Means were declared statistically significant at a 0.05 probability level, or P≤(0.05), using the DUNCAN test (DNMRT).
